# The distribution of Dishevelled in convergently extending mesoderm^☆^

**DOI:** 10.1016/j.ydbio.2013.07.012

**Published:** 2013-10-15

**Authors:** Eleni Panousopoulou, Richard A. Tyson, Till Bretschneider, Jeremy B.A. Green

**Affiliations:** a Department of Craniofacial Development and Stem Cell Biology, Dental Institute, Kings College London, Floor 27 Guy′s Tower, Guy′s Hospital, Great Maze Pond, London, SE1 9RT, UK; b Warwick Systems Biology Centre, University of Warwick, Gibbet Hill Road, CV4 7AL, UK

**Keywords:** Planar cell polarity, Dishevelled, Xenopus, Convergent extension, Localisation, Image analysis, QuimP

## Abstract

Convergent extension (CE) is a conserved morphogenetic movement that drives axial lengthening of the primary body axis and depends on the planar cell polarity (PCP) pathway. In *Drosophila* epithelia, a polarised subcellular accumulation of PCP core components, such as Dishevelled (Dvl) protein, is associated with PCP function. Dvl has long been thought to accumulate in the mediolateral protrusions in *Xenopus* chordamesoderm cells undergoing CE. Here we present a quantitative analysis of Dvl intracellular localisation in *Xenopus* chordamesoderm cells. We find that, surprisingly, accumulations previously observed at mediolateral protrusions of chordamesodermal cells are not protrusion-specific but reflect yolk-free cytoplasm and are quantitatively matched by the distribution of the cytoplasm-filling lineage marker dextran. However, separating cell cortex-associated from bulk Dvl signal reveals a statistical enrichment of Dvl in notochord–somite boundary-(NSB)-directed protrusions, which is dependent upon NSB proximity. Dvl puncta were also observed, but only upon elevated overexpression. These puncta showed no statistically significant spatial bias, in contrast to the strongly posteriorly-enriched GFP-Dvl puncta previously reported in zebrafish. We propose that Dvl distribution is more subtle and dynamic than previously appreciated and that in vertebrate mesoderm it reflects processes other than protrusion as such.

## Highlights

• Bipolar enrichment of Dvl in Xenopus convergently extending mesoderm is non-specific.
• Bulk Dvl co-localises with yolk-free-cytoplasm-filling Dextran.
• Cortical Dvl shows monopolar enrichment in notochord–somite-boundary-directed protrusions.
• Monopolar Dvl enrichment declines with distance from the NSB.
• Dvl accumulation is not absolutely (at least not uniquely) coupled to protrusion.

## Introduction

Convergent extension (CE) is a conserved, early and critical morphogenetic movement that establishes the elongated trunk of the vertebrate body (Keller and Danilchik, 1988). Early embryonic chordamesoderm forms a wide and short tissue, which narrows and elongates anteroposteriorly during and after gastrulation (Keller et al., 1985; Vinson and Adler, 1987). Disruption of CE results in severe gastrulation defects and shortening of the trunk (Ueno and Greene, 2003; Wallingford and Harland, 2001) and can prevent neural tube closure, resulting in an open neural or spina bifida phenotype (Kibar et al., 2001; Ueno and Greene, 2003; Wallingford and Harland, 2001). A similar process describes elongation with concomitant narrowing in development the cochlea (Wang et al., 2005) and other structures, such as kidney tubules (Karner et al., 2009).

CE involves mediolateral cell intercalation (Concha and Adams, 1998; Keller et al., 1989, 1992; Shih and Keller, 1992b). This mechanism has been extensively analysed in *Xenopus* gastrulation (in which such intercalation predominates over other processes such as oriented cell division or medial migration (Keller et al., 2003, 1985)). These studies have revealed that cell intercalation is preceded by multipolar, rapid protrusive activity that slows and becomes selectively mediolateral at midgastrula stage. As protrusions become biased along the mediolateral embryonic axis, the cells progressively elongate in this plane and develop a characteristic morphology with two protruding ends and two longer, relatively smooth anterior and posterior faces. Cells exert traction on one another and intercalate mediolaterally, producing convergence and extension (Keller et al., 1989, 2003; Shih and Keller, 1992a).

Orientation of mediolateral protrusive activity and cell elongation is regulated by the planar cell polarity (PCP) pathway, which was first identified in *Drosophila* as controlling the orientation of structures in the plane of epithelia (Eaton, 1997; Gray et al., 2011; Heisenberg et al., 2000; Wallingford et al., 2000; Wong and Adler, 1993; Zallen, 2007). However, although perturbing the function of core PCP components in *Xenopus* disrupts polarisation of protrusive activity and mediolateral cell intercalation, and thus CE (Darken et al., 2002; Deardorff et al., 1998; Djiane et al., 2000; Goto et al., 2005; Keller and Danilchik, 1988; Wallingford et al., 2000), the mechanism of action of the PCP proteins in this context remains elusive.

PCP core proteins in *Drosophila* epithelia have polarised intracellular distributions, which are required for their function. These proteins include Frizzled (Fz) and Dishevelled (Dsh in *Drosophila*, Dvl in *Xenopus* zebrafish and mammals) enriched on one side of cells, and Prickle (Pk) and Van Gogh (Vang) enriched on the other. The two sides in question include proximal versus distal in wing epithelia, anterior and posterior in larval body epithelium in *Drosophila* (Axelrod, 2001; Strutt, 2001; Usui et al., 1999). Models for PCP in vertebrates have consequently proposed similar PCP protein accumulations in specific cellular quadrants, namely bipolar enrichment in mediolateral protrusions of intercalating cells (Kinoshita et al., 2003; Mlodzik, 2006; Shindo et al., 2008; Wallingford et al., 2002). However, more recently in zebrafish, Dvl has been reported to localise in puncta at the posterior and Pk at the anterior faces of cells in convergently extending chordamesoderm (Ciruna et al., 2006; Yin et al., 2008). However, these studies used heterologous Dvl proteins and described transient and localised Dvl puncta rather than steady-state accumulation. In ascidian notochord, Dvl and Pk colocalise at the mediolateral ends and redistribute to the lateral and the posterior cell edges, respectively, after completion of CE (Jiang et al., 2005). In mouse E12.5 distal limb chondrocytes, Vangl2 protein localises proximally (Gao et al., 2011). Given the importance attached to PCP protein accumulation and the contrasting descriptions of accumulation between *Drosophila* and vertebrates and between *Xenopus* and zebrafish, we performed a detailed quantitative analysis of Dvl intracellular localisation in convergently extending *Xenopus* chordamesoderm. We now report that bulk mediolateral Dvl localisation is non-specific and identical to that of the cytoplasm-filling lineage label dextran. We use new image analysis algorithms to quantifiy Dvl abundance specifically at the cell cortex, where it is generally thought to be active in PCP (Rothbacher et al., 2000; Wallingford et al., 2000; Yang-Snyder et al., 1996), and show that Dvl is accumulated statistically more than dextran in mediolateral cortex, and most conspicuously in the cortex of protrusions directed towards the notochord–somite boundary (NSB), more so in cells just prior to capture by the NSB. We further find that elevating expression (presumably above physiological levels) produces GFP-Dvl puncta but that, in contrast to comparable puncta reported in zebrafish, these have a mild (statistically non-significant) enrichment both posteriorly and at the notochord–somite boundary-directed ends. We conclude that steady-state Dvl distribution does not reflect protrusive activity as such, but possibly NSB-directed migration and perhaps other cellular activities.

## Materials and methods

### mRNA and rhodamine–dextran microinjection

For the cortical analysis of fixed tissues embryos were injected with 100 pg/embryo pCS2Dvl mRNA, 100 pg/embryo pCS2eGFP-CAAX mRNA, and/or 2.5 mg/embryo rhodamine–dextran (Invitrogen, d7163) at the 64 cell stage. For live imaging either 100 or 300 pg/embryo pCS4GFP-Dvl mRNA was injected at the 32 cell stage. DNA constructs were gifts from Sergei Sokol, Vladimir Joukov and Eisuke Nishida, respectively. Embryos were injected in one blastomere at the 32- to 64-cell stage and cultured in 1/10 Normal Amphibian Medium (Sive et al., 2000) to gastrula stages giving mosaic expression in mesoderm.

### Immunolabelling

Gastrula embryos were fixed in MEMFA (Sive et al., 2000) for 1 h, washed 3×15′ in PBT, and blocked for 2 h at room temperature in 10% serum in PBT, incubated overnight at 4 °C with antibodies against c-myc (mouse monoclonal 9E10, Santa Cruz, sc-40), anti-β-catenin (rabbit polyclonal, Santa Cruz, sc-7199), anti-GFP (rabbit polyclonal, Abcam, ab290) followed by secondary antibodies (Alexa, Invitrogen) and mounted in Murray′s Clear (Sive et al., 2000).

### Confocal imaging and image analysis

All imaging was carried out on an inverted Leica SP5 confocal microscope. Laser power, gain and offset were set such that there were no saturated pixels and that all background (determined in uninjected regions of each embryo) was zeroed. Z-stacks from fixed material were captured at 0.5 µm and 2048×2048 pixel (63× objective) resolution with depth compensation. For live imaging, stage 12.5 explants were dissected and cultured in modified Sater′s blastocoel buffer in coverglass-bottomed dishes (Wilson et al., 1989) and explants were imaged every 8 s for 15 min. Image processing and measurements were performed using ImageJ, Fiji, QuimP plugins for Fiji (Bosgraaf et al., 2009; Dormann et al., 2002) and Matlab (MathWorks) for automation and additional quantitation.

## Results and discussion

### Apparent Dvl accumulation at the mediolateral ends of convergent extending chordamesoderm cells is non-specific and coincides with yolk platelet absence

**Fig. 1:**
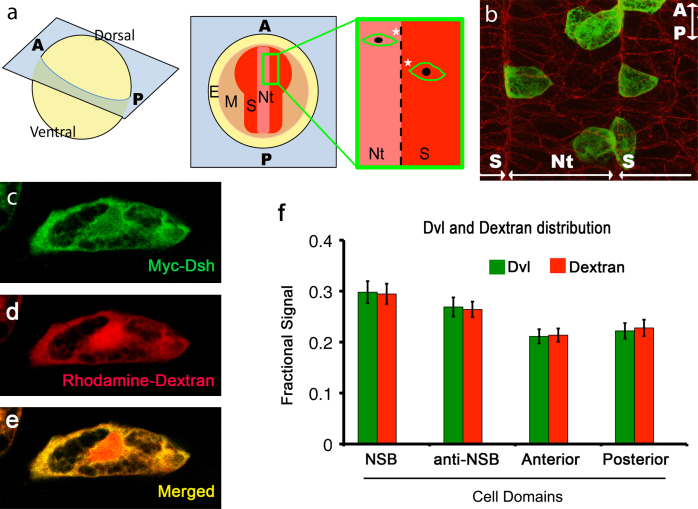
Dvl cytoplasmic distribution in CE chordamesoderm cells is not polarised and is similar to that of dextran. (a) Stage 12.5 embryos were optically sectioned horizontally with respect to the anteroposterior axis, somewhat oblique to the equatorial plane of the gastrula embryo. The NSB runs as two near-parallel lines defining the notochord (Nt) in the middle from somatic mesoderm (S) on either side. All NSB directed ends (asterisks) were grouped versus the anti-NSB directed, anterior (A) and posterior (P). (E) is ectoderm, (M) is mesoderm. (b) A horizontal confocal image of a stage 12.5 embryo showing mosaic expression of myc-Dvl (green cells). beta-catenin staining reveals cellular outlines and the NSB. ((c)–(e)) Colocalisation of Dvl and rhodamine–dextran in a bipolar cell. (f) Bars represent the average fractional fluorescence measurements in the respective cell quadrants (excluding the nucleus) for Dvl and dextran in non-NSB-captured mediolateraly polarised cells at st.12.5. No statistically significant bias in the distribution of the Dvl/dextran ratio was found. Measurements are for 83 cells. Error bars are ±2×standard deviation.

Most previous analyses of *Xenopus* chordamesoderm cells used dorsal marginal zone explants which, while an excellent tool for studying CE, have distorted cells, with a higher length-to-width ratio than the same tissue in vivo (presumably resulting from the mechanical pressure or culture on adherent fibronectin used to keep them flat for observation) (Davidson et al., 2004; Goto et al., 2005). We therefore analysed Dvl intracellular localisation in fixed whole embryos to avoid this distortion. Some previous studies (Kinoshita et al., 2003) have focused on PCP protein distributions at neurulation stages when most chordamesoderm CE due to mediolateral cell intercalation is complete (Keller et al., 1989). We chose to analyse embryos around mid-gastrulation stage (st 12.5), when protrusive activity becomes stabilised in the mediolateral plane and both notochord and pre-somitic mesoderm are still undergoing massive cell intercalation-driven CE (Keller et al., 1989; Keller and Tibbetts, 1989; Shih and Keller, 1992a, 1992b; Wilson and Keller, 1991). Because endogenous Dvl is low-abundance, we used myc-tagged protein, expressed at a level tenfold below the level that produced any phenotypic perturbation (our controls, data not shown). Embryos were injected to give expression in a few isolated cells in the chordamesoderm (Fig. 1b) so that the all-around distribution of the myc-tagged Dvl could be analysed without the ambiguity of signal attribution between abutting labelled cells. Myc-tagged *Xenopus* Dishevelled (Dvl) mRNA was injected in the C1 blastomere (Dale and Slack, 1987) at the 64-cell stage and visualised by fluorescent immunolabelling against the myc tag. We observed that Dvl-associated fluorescence filled the large, stable cytoplasmic protrusions of bipolar chordamesoderm cells, as previously reported (Kinoshita et al., 2003), but also filled the cytoplasm around the nucleus, excluded only from the areas occupied by yolk platelets (Fig. 1c). Yolk platelets are highly dense pararystalline arrays made of vitellogenins absent from cell protrusions such as lamellipodia (Selchow and Winklbauer, 1997). The Dvl distribution we observed suggested that its localisation could reflect yolk exclusion rather than specific recruitment to the mediolateral cell poles. To test this hypothesis, Dvl was coinjected with the biologically inert lineage marker rhodamine–labelled dextran (Gimlich, 1991) and the distributions of the two markers compared. We found that in cells that are not in contact with the NSB, Dvl distribution at stage 12.5 is indistinguishable from that of rhodamine–dextran (Fig. 1c–e), with both occupying yolk-free cytoplasm rather than cell poles specifically. We also performed ratiometric quantitation of Dvl and rhodamine–dextran to confirm colocalisation, ruling out subtle differences not apparent to the eye (Fig. 1f). For this quantitation, we grouped left and right cell ends according to proximity to the notochord–somite-boundary (NSB) rather than “medial” and “lateral” (Fig. 1a), since the convergent extension-related behaviour of chordamesoderm cells at this stage is dependent on boundary capture rather than the embryonic midline (Keller et al., 1992; Shindo et al., 2008). We divided images of each cell into four equal quadrants: anterior, posterior, NSB-directed and anti-NSB-directed and measured the sum of Dvl and dextran-associated fluorescence. The nucleus, marked by dextran, was outlined manually and excluded from the fluorescence measurement (see Supplementary methods for details). We found Dvl and dextran subcellular distributions co-vary such that their ratio has a near uniform distribution. This finding suggests that Dishevelled steady-state, bulk intracellular localisation in cells at this stage is most likely not related to its function in planar cell polarity, contrary to previous interpretations (e.g. Kinoshita et al., 2003; Wallingford et al., 2002).

We also quantified Dvl and rhodamine–dextran distribution in cells captured by the notochord–somite boundary (NSB) (Keller et al., 1989). Dvl signal appeared to be somewhat enriched at the NSB, but as with bipolar cells, this enrichment was also observed for labelled dextran, indicating that these cells have a yolk-free domain at the NSB-directed face where both Dvl and dextran accumulate (Supplementary Fig. 1). In summary, enrichments of Dvl previously observed at mediolateral protrusions of chordamesodermal cells reflect yolk-free cytoplasm and are not specific to cellular protrusions.

Mesoderm is normally considered to be mesenchymal, but it has been suggested that chordamesoderm in *Xenopus* has an “epithelioid” character, given its apparent bilayered structure and seemingly “basal” fibronectin sheath (Davidson et al., 2004; Goto et al., 2005; Green and Davidson, 2007). In *Drosophila* epithelium, PCP proteins are restricted to the peri-apical junctional domains (Axelrod et al., 1998). However, we did not observe any obvious vertically polarised distribution of either Dvl or the basolateral protein PAR-1 (Supplementary Fig. 2).

### Cortical Dvl in Xenopus is slightly enriched in NSB-directed cell cortex

**Fig. 2:**
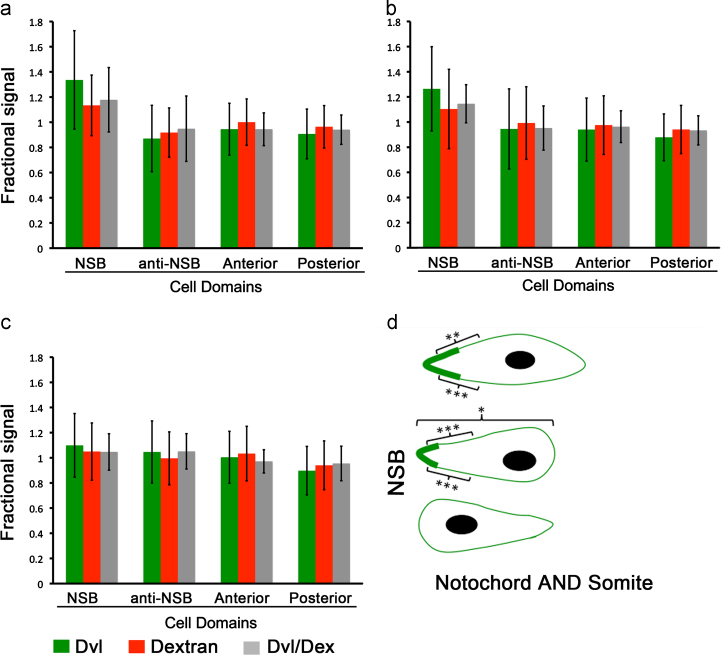
Quantification of Dvl cortical localisation in CE chordamesoderm cells. (a)–(c) Plot of average, fractional fluorescence of Dvl and dextran in the four domains of the cell and the average ratio between Dvl and dextran signal. (a) Non-NSB captured cells bipolarly protruding, towards and away from the NSB (*n*=34). (b) Non-NSB captured cells monopolarly protruding, towards the NSB (*n*=31). (c) Non-NSB captured cells monopolarly protruding, away from the NSB (*n*=24). Error bars represent ±2×standard deviation. (d) Schematic diagram summarising the statistically significant accumulations of Dvl/Dex fluorescence ratio over the entire population of cells analysed. Both bipolarly protruding and NSB-directed protruding cells show Dvl/Dex accumulation in NSB-directed protrusions compared to other cell domains, as indicated. Asterisks represent significance levels for each domain-pairwise comparison, where (^⁎⁎⁎^) represents *p*<0.001, (^⁎⁎^) is *p*<0.01 and (^⁎^) is *p*<0.05. The distances of cells to the NSB are not to scale.

**Fig. 3:**
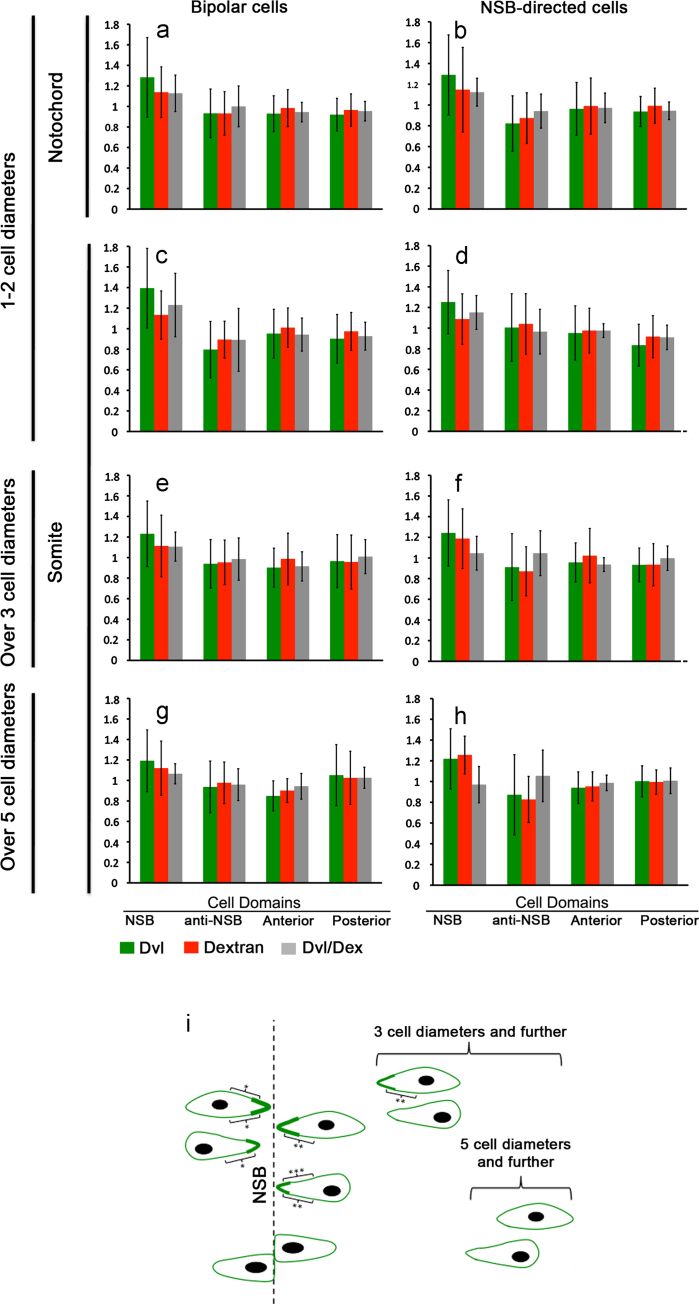
Analysis of Dvl cortical localisation according to cellular location and distance from the NSB. Data from Fig. 2(a) and (b) are here shown broken down accoding to cellular location and distance (in cell diameters) from the notochord–somite boundary. ((a)–(h)) Fractional mean fluorescence of Dvl and dextran and their ratio in each cell domain. ((a)–(d)). Bipolarly protruding ((a) and (c)) and NSB-directed protruding cells ((b) and (d)) within 2 cell diameters from the NSB showing similarly significant enrichment in NSB-directed protrusions of both prospective notochord cells ((a) and (b)) and prospective somite cells ((c) and (d)). Bipolar cells that are 3 or more cell diameters away from the NSB (e) show only a mild enrichment at the NSB directed cell ends and NSB-directed cells (f) show no significant enrichment. Both bipolar and NSB-directed cells 5 or more cell diameters away from the NSB show no enrichment of Dvl in any domain. Error bars are ±2×standard deviation. (i) Schematic diagram summarising the statistically significant accumulations of Dvl/Dex fluorescence ratio in sub-categories of cells as indicated. The thickeness of the green line represents the accumulation of Dvl/Dex fluorescence. Black brackets are representing the pairwise comparisons between cellular domains (using multiple post hoc tests with a Bonferroni correction). Asterisks represent significance levels for each domain-pairwise comparison, where (^⁎⁎⁎^) represents *p*<0.001, (^⁎⁎^) is *p*<0.01 and (^⁎^) is *p*<0.05. The distances of cells to the NSB are not to scale.

In *Xenopus* epithelium, as in *Drosophila*, Dvl is recruited to the cell cortex by PCP signals (Axelrod et al., 1998; Ossipova et al., 2005; Park et al., 2005; Shulman et al., 1998; Yang-Snyder et al., 1996) and membrane localization is essential for PCP signalling (Park et al., 2005). We therefore reasoned that the bulk cytoplasmic distribution described above might be masking a subtle but functional polarised distribution of Dvl at the cell cortex. We measured Dvl fluorescence at the cortex using QuimP, a set of ImageJ plug-ins specifically developed for protein quantitation near cell surfaces (Bosgraaf et al., 2009; Dormann et al., 2002). QuimP uses an active-contour-based method for outlining the cell boundary and enables extraction of fluorescence intensities in the cell cortex, which was here defined as a 5 µm-wide region encompassing the cell membrane and its immediate vicinity. We measured Dvl-associated fluorescence in this region and compared this to rhodamine–dextran fluorescence across the four cell domains described previously. To test for statistical significance in any biased fluorescence accumulations observed we performed ANOVA, with a Greenhouse–Geissner correction, to ensure ANOVA is applied conservatively (avoiding false positive results from datasets with unclear sphericity), followed by multiple comparison post hoc tests with Bonferroni adjustment. We found that upon taking Dvl or dextran alone, there were no significant differences in signal between the four domains. However, upon analysis of the Dvl/dextran ratio, there was a somewhat higher cortical signal in the cell ends that protruded towards the NSB compared to other cell domains (Fig. 2). We observed this NSB-directed Dvl/Dex enrichment not only at the NSB-directed ends of bipolar cells but also at the NSB-directed ends of monopolar cells (Fig. 2a and b). Importantly, monopolar cells protruding away from the NSB showed no significant Dvl/Dex enrichment (Fig. 2c). This latter group provides a critical control that indicates the NSB-dependence of the enrichment. Moreover, it shows that the enrichment is unrelated to the overall level of Dvl overexpression. Grouping the cells by their location in somite or notochord, we additionally observed that the NSB-directed enrichment was present and statistically significant in both medial protrusions of prospective somite cells and lateral protrusions of prospective notochord cells (Fig. 3b and d, summarised in Fig. 3i). This indicates that the NSB itself rather than some more global midline cue is the critical factor in Dvl enrichment. Cells with no protrusions and cells anterior or posterior protrusions showed no significant enrichments (data not shown). NSB-captured cells also showed no significant Dvl/Dex accumulation (Fig. 3i and data not shown).

To determine whether membrane ruffling at protrusive cell ends might contribute to an appearance of accumulated fluorescence we quantified the distribution of a membrane-directed GFP (GFP-CAAX). Although we were unable to analyse GFP-CAAX and Dvl ratiometrically, as the expression of one protein interfered with expression level of the other over a wide range of mRNA injection doses, at the population level, we found no statistically significant biases in GFP-CAAX distribution using ANOVA.

The above results are consistent with previous findings in explants that the NSB can be a tissue orienting cue and that microtubules of chordamesoderm cells become polarised towards NSB-directed cell protrusions prior to boundary capture (Shindo et al., 2008). If the NSB is indeed the critical orienting cue in vivo, one would expect that cells at increasing distances from the NSB should have decreasing Dvl/Dex enrichment. To test this hypothesis we analysed prospective somite cells at varying distances from the NSB. We found that cells up to two cell diameters away from the NSB have the most significant Dvl/Dex NSB-directed enrichment (Fig. 3a–d). Cells at 3 and over cell diameters distance have a much less signfiicant accumulation of Dvl at NSB-directed protrusions (Fig. 3e and f), while cells over 5 cell diameters away from the NSB show no significant accumlation of Dvl/Dex fluorescence in any domain (Fig. 3g and h). Since cells are converging towards the NSB, this implies that in a given cell, NSB-directed cortical Dvl accumulation starts mild and becomes more pronounced as the NSB is approached. The range of this effect (1–3 cell diameters) is reminiscent of a number of reports of signals mediated by transient or extremely fine filopodium-mediated contacts, for example cytonemes in the fly wing, eye and trachea (Roy et al., 2011), “specialised filopodia” in chick limb bud mesenchymal cells (Sanders et al., 2013) and “dynamic filopodia” in the fly notum (Cohen et al., 2010). However, the techniques we used did not reveal any evidence for such structures and they have not been reported by others. An appealing alternative mechanism is mechanotransduction, that is accumulation driven by transduction of mechanical forces generated by differential tissue stiffness along the mediolateral axis, This would be consistent with the length scale not only of the Dvl accumulation but also of of the apparent physical entrainment of cells (making increasingly stable contacts) approaching the NSB (Keller et al., 1989). A similar role for physical force has been reported for the fly wing blade hinge contraction that orients cell polarity over long distances (Aigouy et al., 2010).

The important conclusion from the above results is that while the two ends of bipolar mediolaterally intercalating mesoderm cells cannot be distinguished in terms of protrusive intercalation behaviour (Shih and Keller, 1992a; Wilson and Keller, 1991) they can be distinguished in terms of Dvl accumulation. This suggests that intercalation behaviour is not absolutely (or not uniquely) coupled to Dvl accumulation as such.

### Dvl puncta are dose-dependent and not significantly asymmetrically distributed

**Fig. 4:**
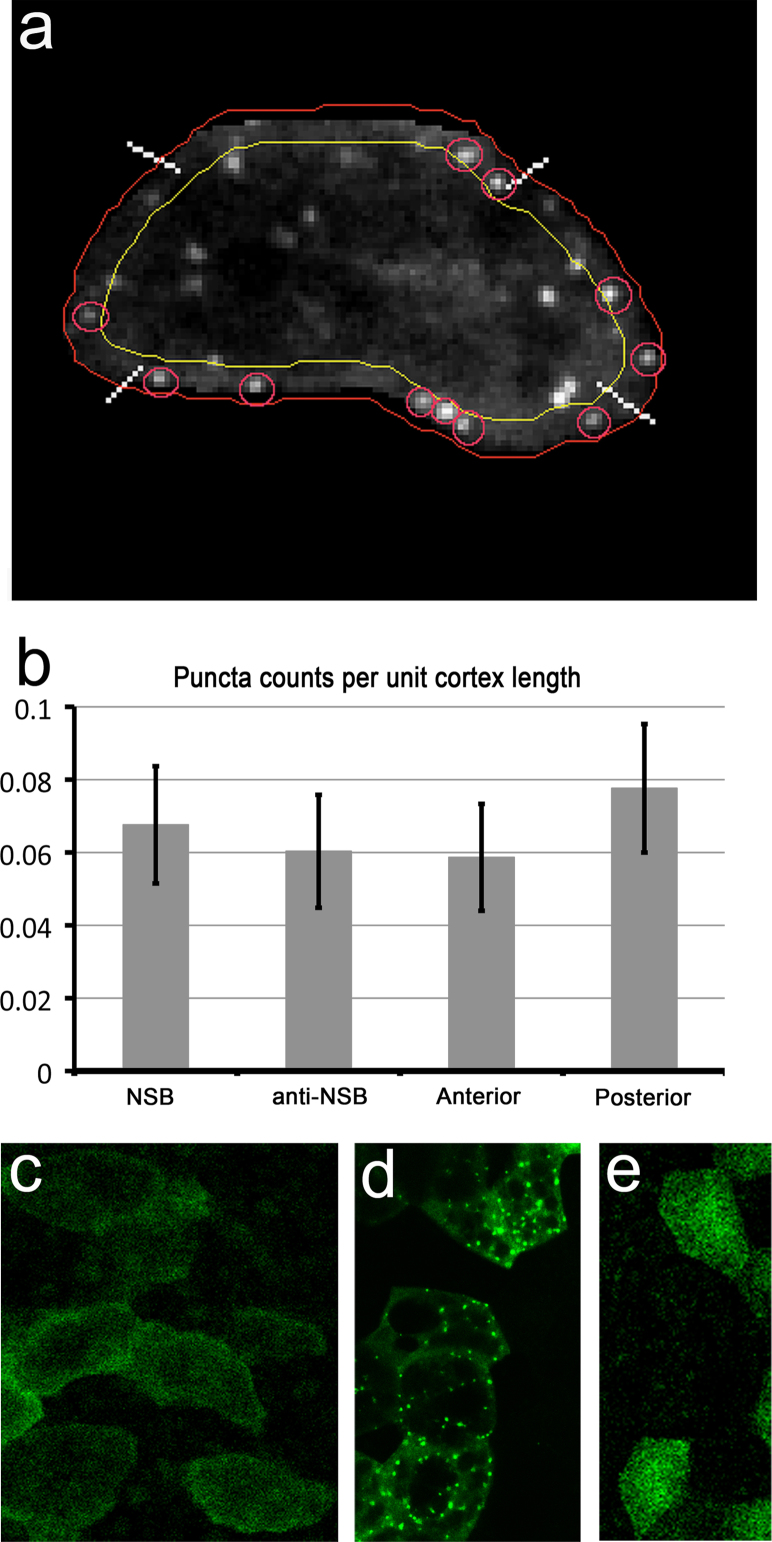
Frequency distribution of GFP-Dvl puncta. (a) Cells expressing GFP-Dvl were segmented as described above (red line around the cell) and the segmentation used to define a cortical strip of 5 μm (defined by outer red line and inner yellow line), which allows for puncta that are cortical, i.e. in very close proximity to the cell edge, to be counted. The cell domains were defined by the diagonals of a bounding rectangle (marked by white lines) and the length of each domain measured. The puncta identified and counted are marked by a red circle. (b) Puncta counts in each domain normalised to cell cortex length showed no statistically significant bias in their distribution. (c) The puncta count for each domain was normalised to the domain length, to account for different contour shapes between domains. No statistically significant bias was found between the four cell quadrants. Error bars are ±2× Standard error of the mean. ((c)–(e)). Controls for live imaging analysis of GFP-Dvl. (c) Single frame from a live stage 12.5 explant expressing 100 pg/embryo GFP-Dvl, showing a weak, diffuse fluorescence. (d) A stage 12.5 explant expressing 300 pg/embryo GFP-Dvl, fixed for 2 h at room temperature in MEMFA and imaged in 30% glycerol/PBS, showing bright cytoplasmic fluorescence and puncta. (e). Single frame from a live stage 12.5 explant expressing 250 pg/embryo of GFP, showing diffuse cytoplasmic fluorescence with no puncta. All injections were done at the 32-cell stage in blastomere C1.

Dishevelled has been reported to localise in the form of transient punctate cortical accumulations in the posterior face of Zebrafish mesoderm cells undergoing CE (Yin et al., 2008), while Prickle localises in a similar cortical punctate fashion at anterior cell faces (Ciruna et al., 2006; Yin et al., 2008). Since we did not observe Dvl puncta in any of the analyses described above, we explored the possibilities that puncta might either be dose-dependent or visible only in live imaging. Injecting a range of doses of GFP-Dvl mRNA, we found that whereas at 100 pg/embryo, used in the experiments above (tenfold below phenotypically perturbing levels of 1 ng per embryo), produced almost no punctate signal, higher doses of 200–300 pg/embryo did produce puncta, both in live imaging and in fixed material in similar numbers and distributions (Fig. 4a and d). Notably, these puncta localised in the cytoplasm as well as around the cell cortex. In live imaging, these puncta were visible for about 1 min on average, although our imaging method could not distinguish whether this was due to instability or simply movement out of the focal plane. We reasoned that despite these puncta being the result of Dvl overexpression, thereby potentially representing non-physiological Dvl localisation, their distribution might still reflect an intracellular bias in Dvl trafficking. To quantify the distribution of puncta around the cell cortex we counted puncta, using QuimP to demarcate the cell cortex as before (Fig. 4a). The puncta counts were normalised to the length of each domain to adjust for different contour shapes. We found that the frequency of puncta is slightly higher in our sample in the NSB-directed and posterior quadrants of the cells but this trend was not statistically significant (Fig. 4b). These data suggest that these Dvl puncta do not reflect a planar polarised pool of Dvl and so may be different from the puncta reported in zebrafish (Yin et al., 2008). In other systems, overexpressed Dvl has been shown to form dynamic aggregates that can be recruited to the cell cortex in response to canonical wnt ligand stimulation (Schwarz-Romond et al., 2005).

Overall, we have shown that Dvl distribution, as marked by epitope-tagged *Xenopus* Dvl at least, is much more subtly polarised that previous reports have suggested: gross bipolar enrichment is non-specific while in the cortex, polarisation is slightly enriched in NSB-directed protrusions. However, this statistical enrichment is not observed in monopolar cells protruding away from the NSB or in protrusions directed away from the NSB in bipolar cells or in elongated cells far away from the NSB. This points to an enrichment correlated with net movement rather than (the bipolar) protrusive behaviour of these cells. The increasing enrichment upon increasing approximation to the NSB is in agreement with this notion.

These data serve as a cautionary note in interpreting protein distribution in yolky embryonic cells and a corrective to the literature regarding bulk localisation. As for Dvl puncta and the differences between *Xenopus* and zebrafish, our dose titration suggests that the *Xenopus* puncta at least are not physiologically normal but are the result of some sort of cellular overload. However, it is likely that they reflect something about the dynamics of Dvl in the cell. The lack of statistical significance of the slight NSB-directed and posterior domain puncta enrichments could in part be due to our sample size, but is clearly much weaker than the overwhelmingly posterior enrichment reported in zebrafish. Their polarised distribution in zebrafish may have something to do with the anteroposteriorly biased cell divisions in that species (Concha and Adams, 1998) since cell division does redistribute PCP components in other contexts (Devenport et al., 2011) while in *Xenopus* gastrula mesoderm cell division is actively suppressed (Shih and Keller, 1992a, 1992b).

High resolution studies of other PCP components’ dynamic localisation and/or development of an in situ assay for PCP protein “activity” are required if we are to gain insight into how PCP organises tissue. Meanwhile, development of better image analysis tools for quantification of the spatial distribution of molecular events remains an important non-trivial task for future studies.

## Footnotes

Appendix A Supplementary data associated with this article can be found in the online version at doi:10.1016/j.ydbio.2013.07.012.
