# Supplementary material for: The distribution of Dishevelled in convergently extending mesoderm
Source: Dev Biol. 2013 Oct 15;382(2):496–503. doi: 10.1016/j.ydbio.2013.07.012 (PMC3793869; doi:10.1016/j.ydbio.2013.07.012)
Supplement: Supplementary file 1 — Supplementary data [file mmc1.pdf]

Panoousopoulou et al.

Supplementary Methods

Definition of four equal cell quadrants for image analysis.

Keller lab observations over many years have shown that the medial and lateral ends of bipolar cells behave the same in mediolateral intercalation (Keller and Tibbetts, 1989). However, the notochord has been shown to play a role as an orienting cue (Shindo *et al.*, 2008). Therefore, the entire analysis is done with the NSB as a point of reference, with the four cellular domains quantified for Dvl fluorescence being: NSB-directed cell ends, anti-NSB, anterior and posterior.

In order to quantify bulk cytoplasmic fluorescence, we wished to measure the sum of Dvl and Dextran-associated fluorescence across these four domains. Therefore, we divided cells into four quadrants of equal area, that coincided with the four cardinal directions.

Commonly, the diagonals of a bounding rectangle are used to divide chordamesoderm cells into mediolateral and anterioposterior domains to quantify direction of lamelliform protrusions, for instance (Kinoshita *et al.*, 2003). However, this method does not give quadrants of equal area. We therefore performed the following steps. (Figure M1). We used ImageJ to determine the cell geometrical centroid by hand-drawing around the cell image and then using the command “Measure” under the “Analyse” menu, which returns the geometrical centroid among other parameters of the outline shape (figure M1, b). The nucleus was traced by hand and blacked out (figure M1, a). Because this centroid is the average of the x and y coordinates of the pixels in the outlined shape (effectively the shape’s centre of mass), any line through the centroid divides the xy shape into two equal areas (figure M1, c). Such a line was drawn at a 45° angle to the AP axis. Rather that draw a second line through the centroid at right-angles to the first, the centroids of the two halves were found using ImageJ on each, and a line passing through each of these centroids was drawn perpendicular to the first line (figure M1, d). This latter method divides the cell image into four quadrants of equal area in pixels, unlike a second line at right-angles to the first (which generates unequal areas).

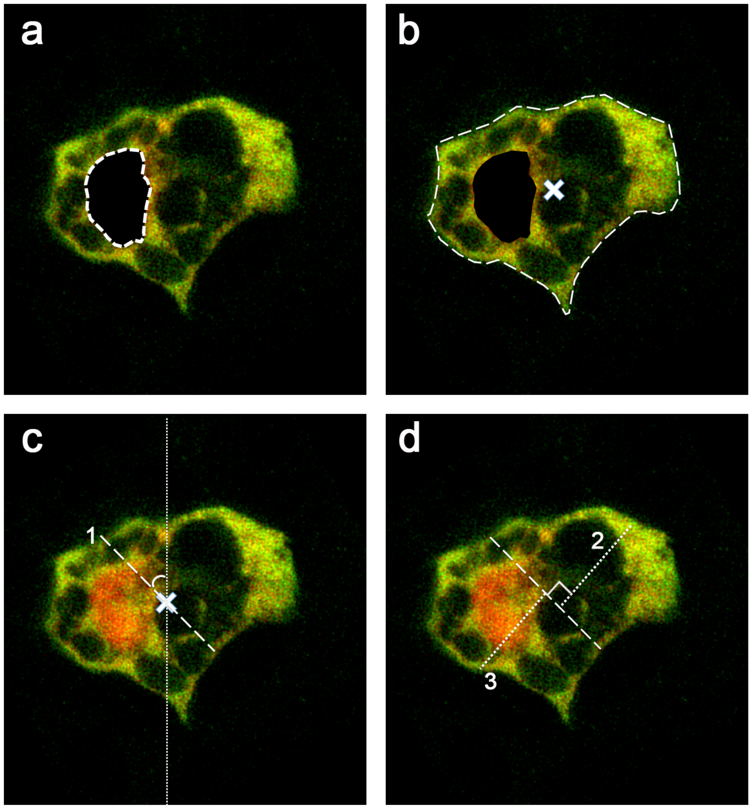

Figure M1. Division of chordamesoderm cells in four equal quadrants for cytoplasmic Dvl localisation alanysis.

a. A hand-drawn selection area was drawn around the nucleus (white dotted line), which was then colored black so as not to contribute to fluorescence measurements. **b.** A second selection area was then drawn around the cell (white dotted line), such that the centroid (white x) can be determined, as the average of all x and y distances of the cell. **c.** The first line (1) was then drawn that passed though the centroid and was 45 degrees (white arc) to the AP axis, dividing the cell into two equal areas. **d.** The centroids of the two cell halves were determined in the same way as described above and the second and third lines (2 and 3) were then drawn such that they passed through the respective centroids and were perpendicular to the first line.

Analysis of cortical Dvl distribution.

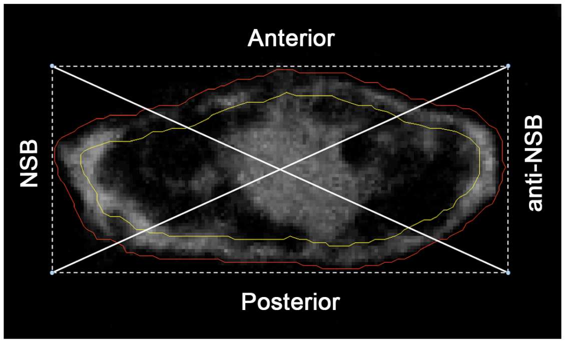

Analysis of cortical intensity was done using the QuimP11 software package. Individual cells were segmented using QuimP’s active contour and the cortex defined as a 5 micron strip around the cell perimeter. For each point on the perimeter, QuimP's ANA plugin was used to measures the maximal cortical Dvl intensity across the width of the cortex (the use of maximal fluorescence, rather than total cortical intensity, prevented inaccuracies in segmentation and positioning of yolk granules affecting measurements). Dextran intensity was then measured at the same locations as Dvl sampling, hence providing a true ratio. Using QuimP's MATLAB scripts, the cell perimeter was then divided into the four domains corresponding to the embryonic axes. For this analysis domain boundaries were defined by the corners of a bounding rectangle.

For each cell, the sum of sampled intensities (for either Dvl, Dextran, or GFP-CAAX) was determined for individual domains and converted to fractional measurements by dividing with each cell’s total fluorescence. The resulting proportions were then corrected for the area/perimeter lengths of the domains, and domain averages computed for all cells. To produce a ratio of Dvl to Dextran, prior to averaging the proportion of Dvl in a domain was divided by that of Dextran and the log taken as to produce positive values for Dvl enrichment, and negative values for Dextran enrichment.

Live imaging of GFP-Dvl expressing chordamesoderm of stage 12.5 explants.

Explants were prepared from stage 12.5 GFP-Dvl expressing embryos by dissecting out the dorsal roof and peeling away the endoderm in order to expose the mesoderm, as described in (Wilson et al., 1989).

Laser penetrative power allowed us to image 15-20 microns deep, covering most of a cell layer. We wished to be confident that we were analyzing images that represented the z-midplane and not grazing sections. To this end, we performed live imaging on a 2 photon confocal microscope, where penetrative power was over 50μm, confirming that we were not obtaining grazing optical section. This imaging was done with the kind assistance of Dr. Fred Festy (Biomaterials, Tissue Engineering & Imaging, King's College London).

Kinoshita, N., H. Iioka, A. Miyakoshi and N. Ueno (2003). PKC delta is essential for Dishevelled function in a noncanonical Wnt pathway that regulates Xenopus convergent extension movements. *Genes Dev* **17**(13), 1663-76.

Shindo, A., T. S. Yamamoto and N. Ueno (2008). Coordination of cell polarity during Xenopus gastrulation. *PLoS One* **3**(2), e1600.

Wilson, P. A., G. Oster and R. Keller (1989). Cell rearrangement and segmentation in Xenopus: direct observation of cultured explants. *Development* **105**(1), 155-66.
