# Supplementary material for: The distribution of Dishevelled in convergently extending mesoderm
Source: Dev Biol. 2013 Oct 15;382(2):496–503. doi: 10.1016/j.ydbio.2013.07.012 (PMC3793869; doi:10.1016/j.ydbio.2013.07.012)
Supplement: Supplementary file 2 — Supplementary data [file mmc2.pdf]

Panousopoulou *et al*.

Supplementary Figures

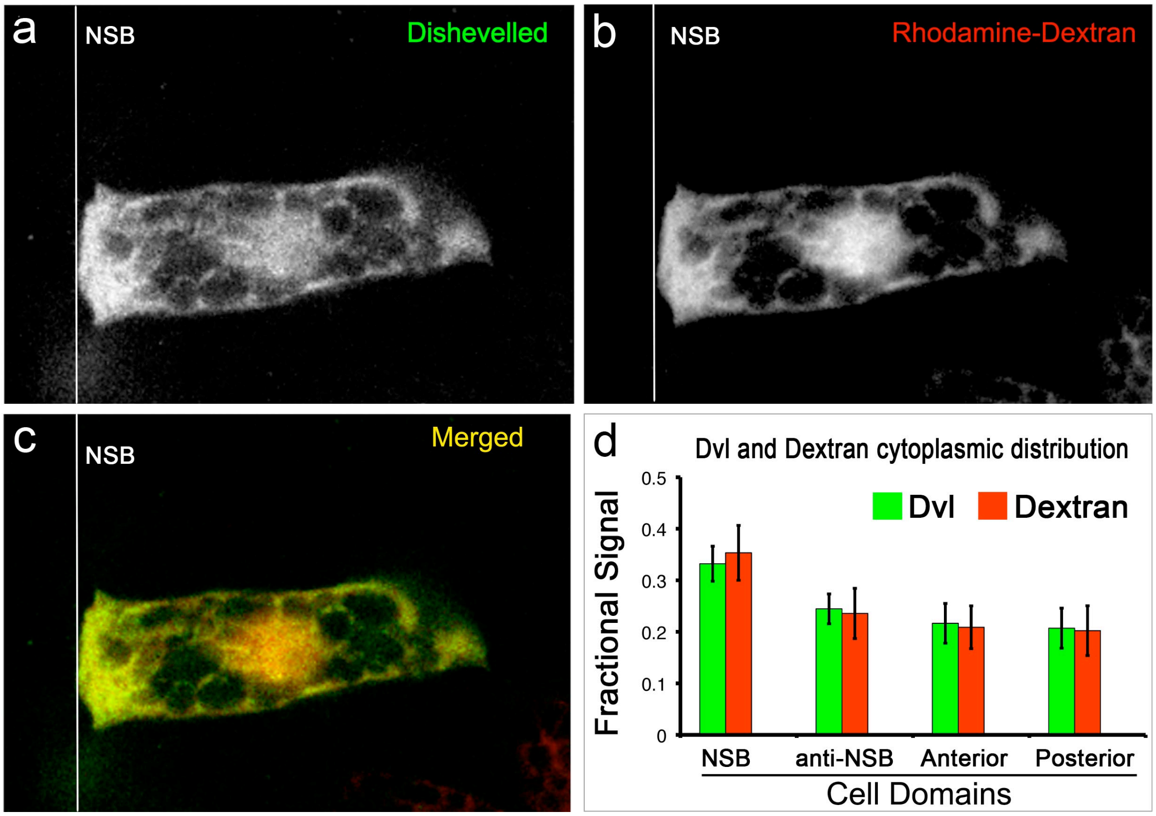

Supplementary Figure 1. Dishevelled and Dextran show the same type and amount of enrichment at the NSB.

(a-c). A sample of an NSB-captured cell expressing Dvl ("a", green channel) and Dextran ("b", red channel). The white line in "c" (merged channels) marks the NSB. (d) Average, fractional fluorescence measurements of Dvl (green columns), Dextran (red columns) and the log of the Dvl/Dextran fluorescence ratio. Error bars are +/- 2 x standard error of the mean.

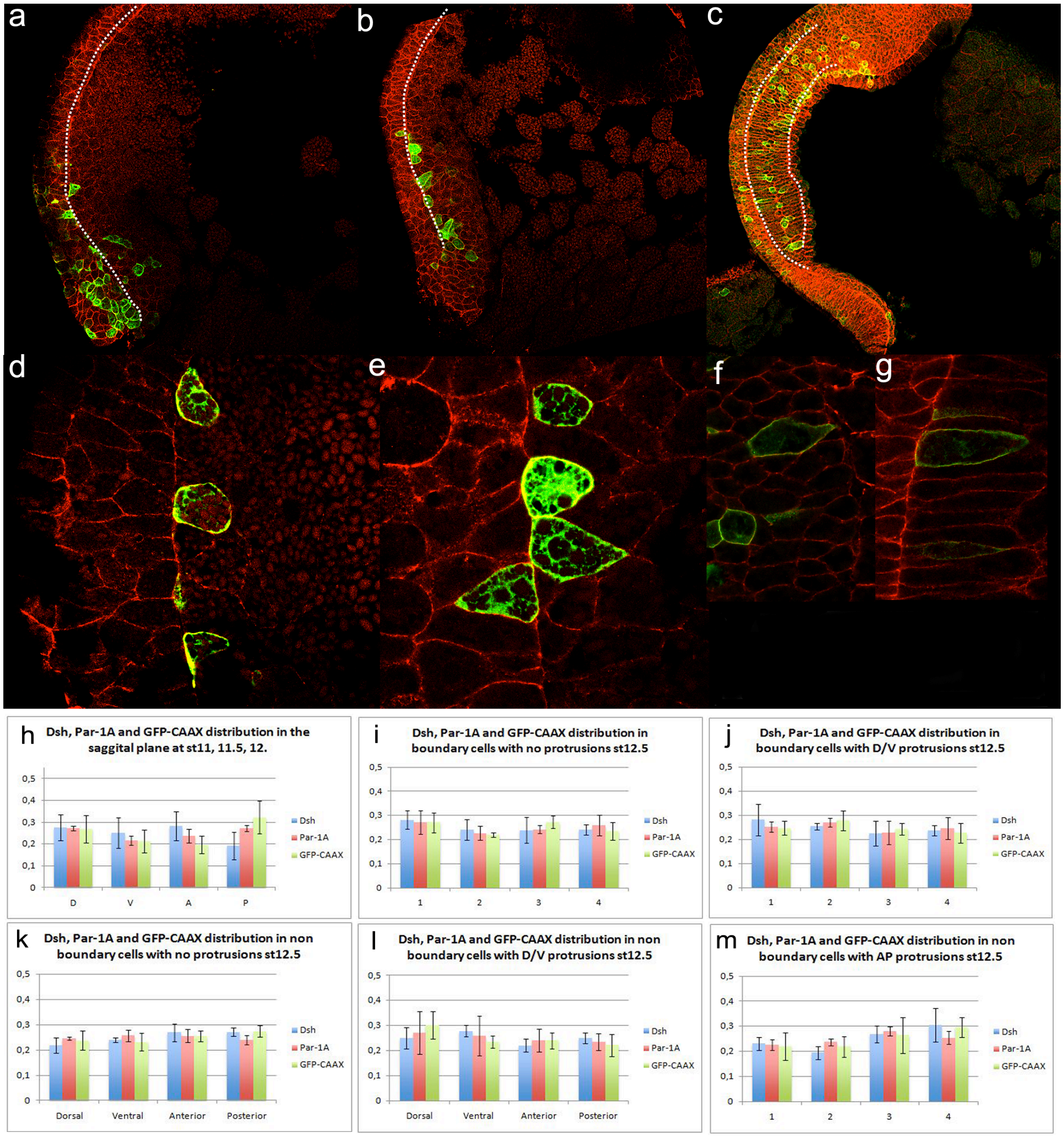

Supplementary Figure 2. Distribution of Dishevelled in the sagittal plane throughout stages 11 to 12.5.

Anterior is to the top, red staining is anti-b-catenin (Alexa 568 secondary antibody), green staining is anti-myc-Dvl (Alexa 488 secondary antibody). (a) a x20 magnification of a sagittal view of a stage 11 embryo. Scale bar is 100μm. White dotted lines outline the boundaries between the ectoderm and mesoderm in stages 11.5 and 12 and between mesoderm and endoderm in stage 12.5. (d) A x 63 magnification of a sagittal view of a stage 11 embryo showing individual

myc-Dvl expressing mesoderm cells. Scale bar is 100μm. The dotted and dashed white lines outline the same tissue boundaries as in (a). (b) x20 magnification of a stage 12 embryo, where the white dotted and dashed lines depict the same tissue boundaries as in (a). (e) is a x63 magnification of a st12 embryo showing one ectodermal and three mesodermal myc-Dvl positive cells. (c) x20 of a late st12.5 embryo, where the dashed white lines depict the dorsal and ventral mesoderm boundaries and (f) and (g) are x63 magnifications of the same stage as (c), showing an example of non-boundary touching (f) and a boundary touching (g) myc-Dvl expressing cells. (h-m): Histogram bars represent the averaged fractional Dishevelled, Par-1A and GFP-CAAX measurements in each of the four cell measured domains (dorsal, ventral, anterior, posterior). Error bars are standard deviations intracellular distribution of Dishevelled (blue bars), Par-1A (red bars) and GFP-CAAX (green bars) in sagittal view, from stage 11 to 12.5. (h) The distribution of Dvl, Par-1A and GFP-CAAX through stages 11, 11.5 and 12 has all been summarised, as they were very similar (whether cells were touching or not the dorsal boundary). For stage 12.5 cells that form part of the boundary (i, j) are presented separately than those that don't (k, l, m). Again, no significant enrichment in any cell domain or direction is observed for Dishevelled or Par-1A and the distribution of both proteins is highly nearly indistinguishable from GFP-CAAX.
